# Transcranial magnetic stimulation over the right dorsolateral prefrontal cortex modulates visuospatial distractor suppression

**DOI:** 10.1111/ejn.15164

**Published:** 2021-03-17

**Authors:** Zhenlan Jin, Ke Xie, Xuejin Ni, Dong‐Gang Jin, Junjun Zhang, Ling Li

**Affiliations:** ^1^ Key Laboratory for NeuroInformation of Ministry of Education High‐Field Magnetic Resonance Brain Imaging Key Laboratory of Sichuan Province School of Life Science and Technology University of Electronic Science and Technology of China Chengdu China

**Keywords:** distractor suppression, dorsolateral prefrontal cortex, location, saliency, transcranial magnetic stimulation

## Abstract

Visual selective attention allows us to filter relevant inputs from irrelevant inputs during visual processing. In contrast to rich research exploring how the brain facilitates task‐relevant inputs, less is known about how the brain suppresses irrelevant inputs. In this study, we used transcranial magnetic stimulation (TMS) to investigate the causal role of the right dorsolateral prefrontal cortex (DLPFC), a crucial brain area for attentional control, in distractor suppression. Specifically, 10‐Hz repetitive TMS (rTMS) was applied to the right DLPFC and Vertex at the stimuli onset (stimuli‐onset TMS) or 500 ms prior to the stimuli onset (prestimuli TMS). In a variant of the Posner cueing task, participants were instructed to identify the shape of a white target while ignoring a white or colored distractor whose location was either cued in advance or uncued. As anticipated, either the location cue or the colored distractor led to faster responses. Notably, the location cueing effect was eliminated by stimuli‐onset TMS to the right DLPFC, but not by prestimuli TMS. Further analyses showed that stimuli‐onset TMS quickened responses to uncued trials, and this TMS effect was derived from the inhibition at the distractor in both visual fields. In addition, TMS over the right DLPFC had no specific effect on the colored distractor compared to the white one. Considered collectively, these findings indicate that the DLPFC plays a crucial role in visuospatial distractor suppression and acts upon stimuli presentation. Besides, it seems the DLPFC contributes more to location‐based distractor suppression than to color‐based one.

## Graphical abstract

The location cue or salience of a distractor triggers distractor suppression. Stimuli‐onset DLPFC TMS enhances distractor suppression triggered by location cue only. No prestimuli DLPFC‐TMS effect is found on distractor suppression.

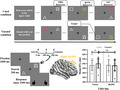

**Abbreviations**
adjRTs: adjusted reaction times
DLPFC: dorsolateral prefrontal cortex
LVF: left visual field
rTMS: repetitive transcranial magnetic stimulation
RTs: reaction times
RVF: right visual field
TMS: transcranial magnetic stimulation

## 1 INTRODUCTION

Amid a sea of incoming stimuli, human observers must filter relevant from irrelevant information during visual processing (Awh et al., 2003; Gazzaley et al., 2007; Posner, 1980). According to the biased competition model, a number of candidates compete for limited attentional resources, and only the winner can enter into visual working memory and consciousness (Desimone & Duncan, 1995). During this competition, bottom‐up factors (e.g., inherent saliency of stimuli) can automatically capture limited attentional resources (Belopolsky et al., 2007; Franconeri & Simons, 2003; Theeuwes, 2010). Additionally, top‐down factors (e.g., information of target locations) can voluntarily drive attention toward task‐related stimuli and away from irrelevance (Chao, 2010; Noudoost et al., 2010; Theeuwes, 2010). For example, subjects' behavioral performance can be highly improved if the target location is cued in advance (Posner, 1980; Posner et al., 1980). In other words, advanced information of spatial location endows the target with selection priority and enhances processing at the cued location (i.e., target facilitation).

As an important counterpoint to target facilitation, top‐down suppression of task‐irrelevant information equally contributes to enhancement of relevant input (Gaspelin & Luck, 2018; Noonan et al., 2018; Zanto & Rissman, 2015). It has been proposed that inhibitory neural dynamics likely play an important role in shaping information processing (Jensen & Mazaheri, 2010; Markram et al., 2004). Numerous studies demonstrated that foreknowledge of a potential distractor location could efficiently quicken subjects' responses via an active inhibition of the cued location (Chao, 2010; Munneke et al., 2008; Ruff & Driver, 2006). Similarly, in an eye movement study, subjects' eye movement deviated away from the cued distractor location, directly confirming that the distractor location was actively inhibited based on top‐down expectancy of where the distractor would appear (Van der Stigchel & Theeuwes, 2006). In addition, salient‐but‐irrelevant singletons, traditionally impairing visual search from attention capture, can in turn benefit task performance if their identities (e.g., colors) are known in advance (Gaspelin et al., 2015, 2017). These findings provide evidence for the idea that prior knowledge (e.g., location, color) of the distractor can facilitate target processing by actively inhibiting distractor processing.

Neuroimaging studies investigating the neural basis of attentional control have revealed that top‐down modulation signals primarily arise from the frontoparietal network including the prefrontal cortex (Barceló et al., 2000), frontal eye fields (Sylvester et al., 2008), and posterior parietal cortex (Kanai et al., 2011). Among these, increased activation of the dorsolateral prefrontal cortex (DLPFC), a hub of the frontoparietal network, has been observed during various attentional control tasks, such as inhibitory control, conflict resolution, and spatial priming (Diamond, 2013; Egner & Hirsch, 2005; Krueger et al., 2007; MacDonald et al., 2000). Also, a study by Toepper et al., (2010) found increased activity of the DLPFC when distractors appeared during the encoding phase of spatial working memory, indicating the involvement of left DLPFC in distraction suppression during spatial working memory encoding. Furthermore, Suzuki and Gottlieb (2012) observed increased distractibility when the DLPFC neurons were inactivated in a memory‐guided saccade task where a salient distractor flashed unpredictably, highlighting the significance of the DLPFC in distractor suppression. Another study using a negative priming paradigm observed higher right DLPFC activation when a target appeared at a previously inhibited location compared with that when a target appeared at other locations (Krueger et al., 2007). Supportively, TMS over the right DLPFC suppressed the negative priming when a single pulse TMS was applied 100 ms after the probe display onset (Kehrer et al., 2015). These results suggest a crucial role of the DLPFC in distractor suppression. Generally, visuospatial attention has been consistently demonstrated hemispheric lateralization (Bartolomeo & Seidel Malkinson, 2019; Jansen et al., 2004). More specifically, there is a right hemispheric dominance of the dorsal frontoparietal network (FPN) in suppressing salient‐but‐irrelevant distractors (Hodsoll et al., 2009; Lega et al., 2019). Therefore, it is of great interest to ascertain the causal role of the right DLPFC, a crucial hub of the FPN, in distractor suppression.

Transcranial magnetic stimulation (TMS), a non‐invasive brain stimulation technique, uses brief high‐intensity magnetic field pulses to regulate cortical excitability (Kozyrev et al., 2014) and is widely used to examine brain–behavior relationships across multiple cognitive functions (Beynel et al., 2019; Lega et al., 2019; Yamanaka et al., 2010). In particular, the online rTMS protocol applied at specific time points during a task provides insight to explore the exact timing of neural processing (Beynel et al., 2019; Kehrer et al., 2015; Luber et al., 2007). Thus, the current study used online rTMS to explore the contribution of the right DLPFC to distractor suppression. Specifically, a cue could validly indicate the upcoming location of the distractor in an attempt to trigger an active inhibition of the cued location, and no location cue was provided (Noonan et al., 2016). In addition, the saliency of distractors was manipulated to produce salient distractors to trigger feature‐based suppression (Gaspelin et al., 2015, 2017). The rTMS (3‐pulses, 10 Hz) was applied to the right DLPFC or Vertex at the stimuli onset or 500 ms prior to the stimuli onset, respectively. The primary purposes of this study are to: (a) examine the causal role of the right DLPFC in distractor suppression which was recruited by foreknowledge of distractor location or distractor salience, and (b) ascertain the temporal contribution of the right DLPFC to this top‐down mechanism. We hypothesized that the rTMS over the right DLPFC would regulate distractor suppression.

## 2 MATERIALS AND METHODS

### 2.1 Participants

Healthy, right‐handed subjects with normal or corrected‐to‐normal vision were recruited from the University of Electronic Science and Technology of China (UESTC). There were sixteen subjects in Experiment 1 (7 females; mean age ± *SD*, 21.94 ± 2.11 years) and in Experiment 2 (8 females, mean age ± *SD*, 21.69 ± 2.02 years), respectively. All subjects were unaware of the purposes of the study. There were no subjects participating in the two experiments. We obtained each subject's written informed consent in advance. The current research was in accordance with the Helsinki declaration and was approved by the UESTC Institutional Review Board.

### 2.2 Stimuli and procedures

**FIGURE 1:**
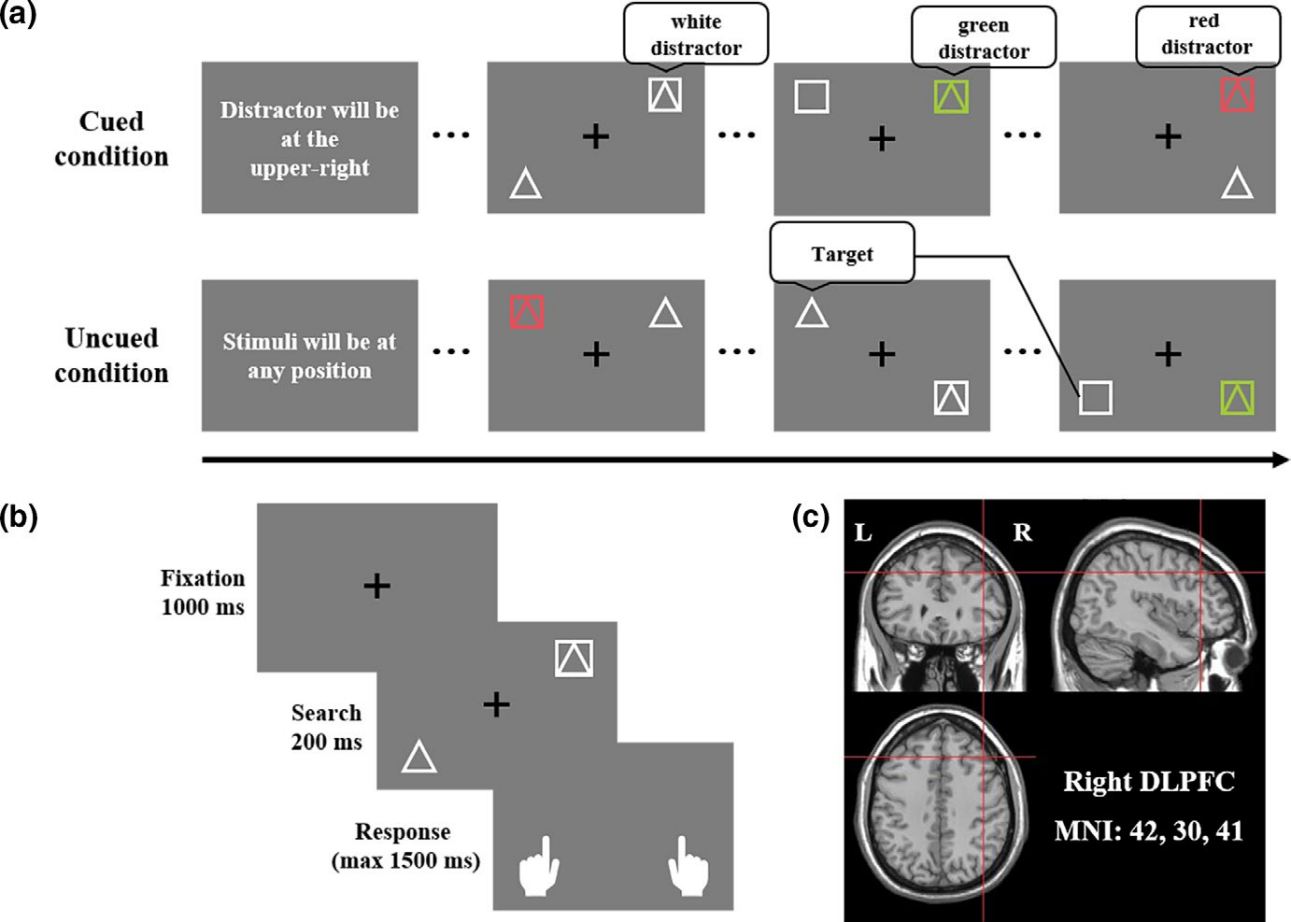
Experimental design and procedure. (a) The experiment was designed in blocks, and each block began with an instruction display indicating the forthcoming task. (b) Each trial was started with a fixation display to fix the subject's attention. Following a brief delay (1,000 ms), a stimuli display was presented for 200 ms, which contained a target to be identified and a distractor to be filtered out. Subjects were instructed to quickly identify the shape of the target by pressing a button. (c) The location of the right dorsolateral prefrontal cortex (MNI coordinate: *x*, *y*, *z* = 42, 30, 41)

Stimuli presentation and response registration were controlled via a PC with E‐Prime 2.0 (Psychology Software Tools) equipped with a 1,366 × 768 pixels resolution, 60 Hz monitor viewed from a distance of 55 cm. Visual search arrays consisted of a black central fixation cross (2.3 degree, RGB, 0 0 0), a white target [RGB, 240 240 240], and a distractor. Each item subtended a visual angle of 2.3° by 2.3°. All stimuli were presented at one of four locations forming an imaginary square, with the eccentricity of 7.0° from the central fixation point (Figure 1a). All stimuli were presented on a gray background [RGB, 125 125 125]. The target was either a triangle or a square, and the distractor was the two targets superimposed in white or colored. The target and distractor colors varied and followed these display configurations: white target and distractor (50%); white target, red distractor [RGB, 230 80 90; 25%]; white target, green distractor [RGB, 160 200 60; 25%]. These display configurations were randomly intermixed across trials.

This study included two conditions: distractor cued condition (Figure 1a, top panel) and distractor uncued condition (Figure 1a, bottom panel). On the distractor cued condition, subjects would be given the distractor location at the beginning of a block in writing to trigger distractor suppression. Hence, the distractor was fixed, and the target would appear at one of the remaining three locations equally. On the distractor uncued condition, there was no spatial information prior to a block. In this case, the target and distractor would be presented at any position of the four candidates. The time course of a trail was illustrated in Figure 1b, a search display in each trial was presented after a 1,000 ms fixation period marked by a black central fixation cross to aid steady fixation. Next, the search display remained visible for 200 ms and was followed by a blank display with a maximum duration of 1,500 ms. Subjects were instructed to judge the shape of the target by pressing “1” for a triangle target and “2” for a square target, respectively. Each subject performed two sessions (one session per condition) of four blocks (24 trials per block), and were encouraged to take a break between blocks and continued when ready. Each subject also performed two practical sessions prior to experimental sessions. The order of the sessions was balanced across the subjects, and the order of four blocks within a session was randomized as well.

### 2.3 TMS protocol and stimulation sites

A Magstim super rapid stimulator equipped with a prevalent 70‐mm figure‐8 coil was used to deliver TMS (Magstim Company Ltd). Landmarks on the participants' heads were co‐registered to individual structural magnetic resonance images via the BrainSight stereotaxic system (Frameless, Rogue Research). More specifically, BrainSight utilized optical tracking to locate the position of the TMS coil throughout the stimulation period, ensuring the TMS coil remained on the stimulation sites with a precision below 0.5 mm. Also, structural magnetic resonance images were acquired using a GE Sigma 3.0‐Tesla scanner (General Electric) at the UESTC with the following parameters: TR = 5.96 ms, TE = 1.96 ms, FA = 9°, FOV = 256 × 256 mm^2^, voxel size = 1 × 1 × 1 mm^3^, 176 slices, slice thickness/gap = 1.0/0 mm).

In this study, online repetitive transcranial magnetic stimulation (rTMS, 3 pulses at 10 Hz) was delivered to the Vertex and right DLPFC. This protocol has been proved to effectively modulate cortical activity (Lega et al., 2019; Saad & Silvanto, 2013). The target site of the right DLPFC (Figure 1c; MNI coordinate, *x*, *y*, *z* = 42, 30, 41) was proposed to be related to distractor processing (Kehrer et al., 2015; Krueger et al., 2007). As the control site, the Vertex was located at the middlemost location of the head (Kalla et al., 2009; Kiyonaga et al., 2014; Wang et al., 2018). For the intensity of the rTMS, it is generally set relative to the motor threshold with the assumption that excitability in non‐motor areas is similar to that of motor cortex (Ruohonen & Ilmoniemi, 2002; Stokes et al., 2005, 2007). However, studies showed that motor threshold is not necessarily a reliable indicator of cortical excitability of other brain regions (McConnell et al., 2001; Rushworth et al., 2001; Stewart et al., 2001). Thus, this study picked a fixed intensity of 30% of the maximum stimulator.

**FIGURE 2:**
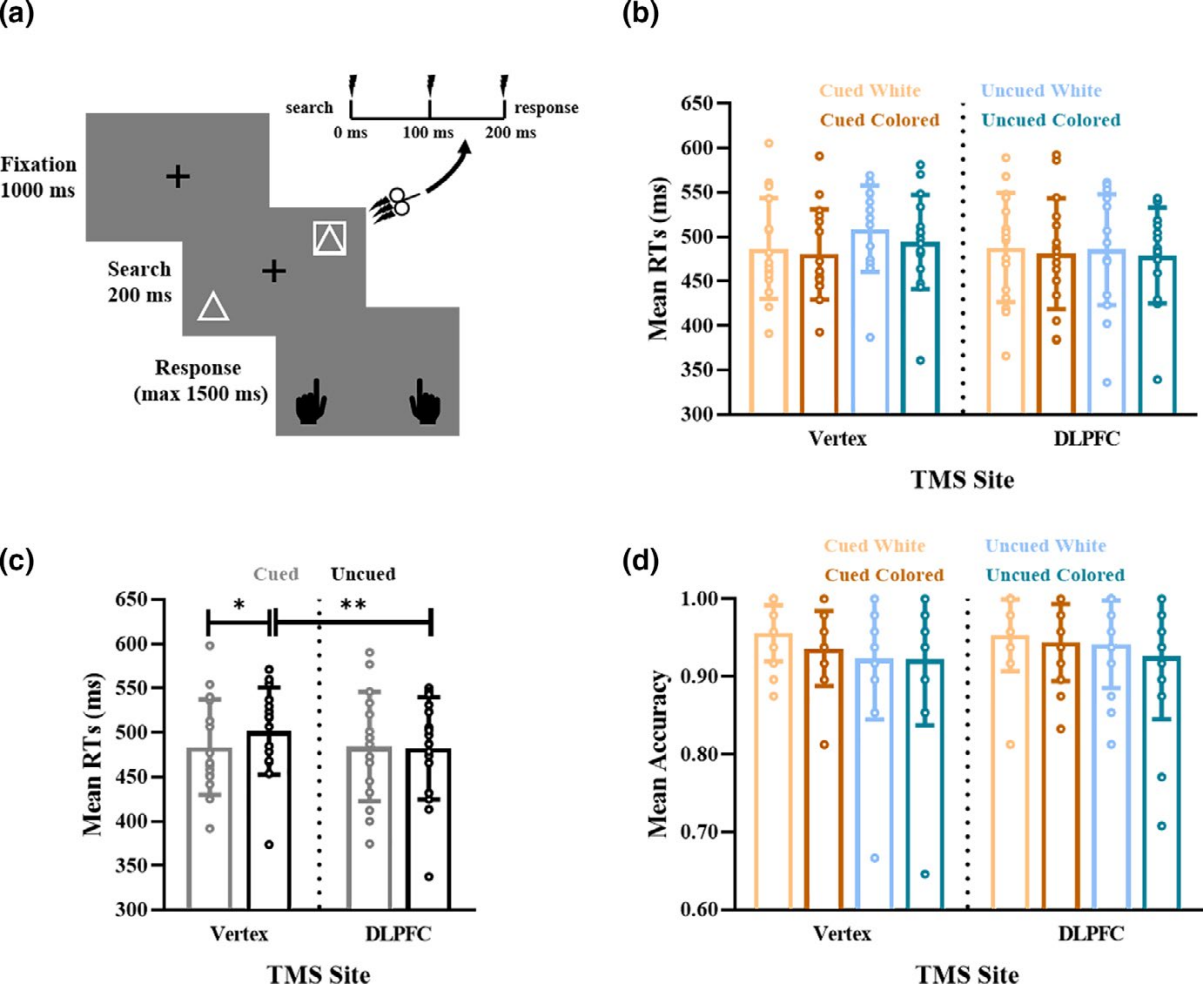
Time course of the visual search task and behavioral performance in Experiment 1. (a) rTMS was applied to the right dorsolateral prefrontal cortex (DLPFC) or Vertex at the search stimuli onset. (b) The mean reaction times (RTs) per condition. (c) The mean RTs with right DLPFC and Vertex TMS in the cued and uncued conditions. (d) The mean accuracy per condition. Error bars represent the standard deviation of the means. *p <.05, **p <.01, ***p <.001

**FIGURE 3:**
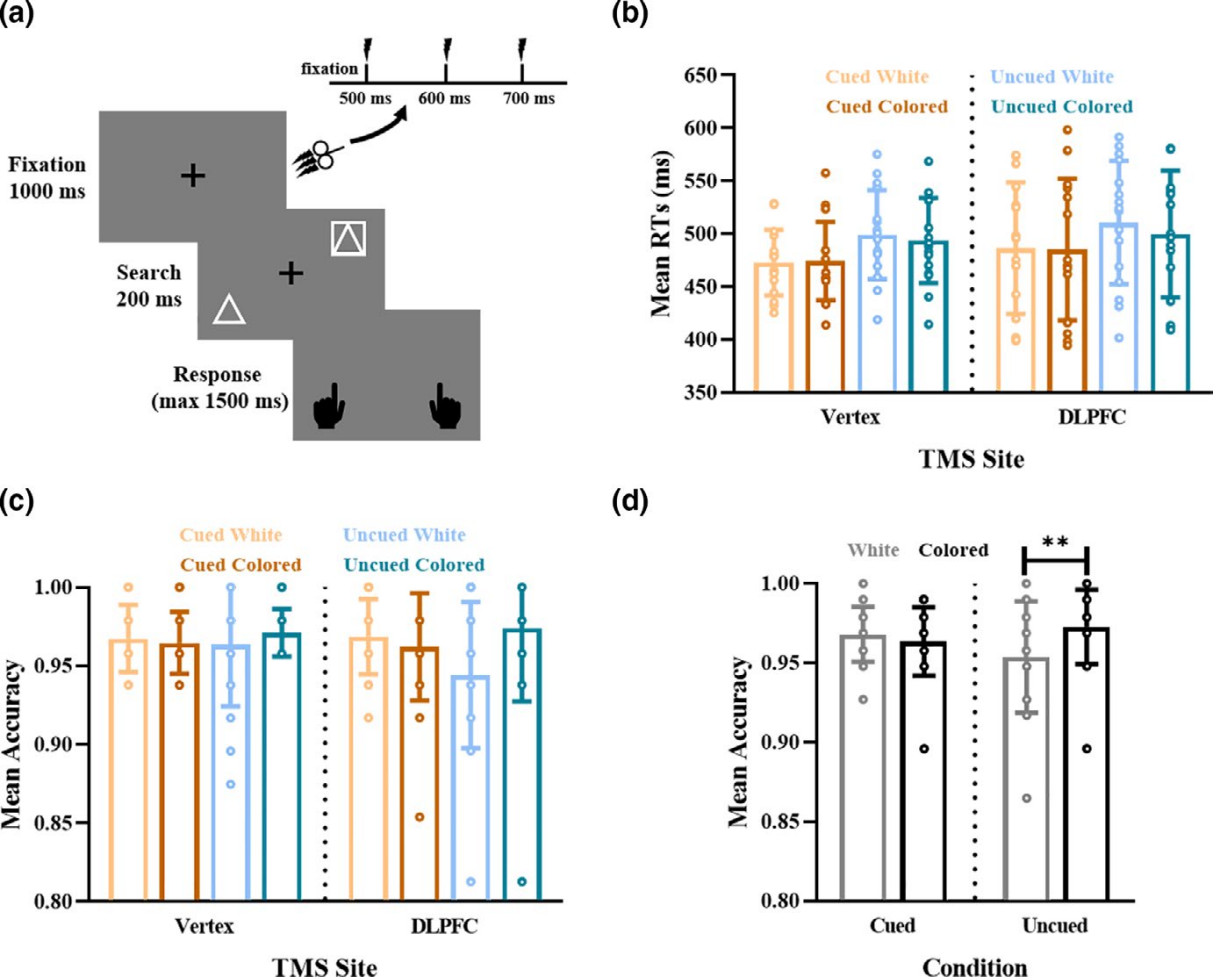
Time course of the visual search task and task performance in Experiment 2. (a) rTMS was applied to the right dorsolateral prefrontal cortex or Vertex 500 ms prior to the search stimuli onset. (b) The mean reaction times (RTs) and accuracy (c) per condition. (d) The accuracy was plotted for trials in which the distractor was white or colored in the cued and uncued condition, respectively. Error bars represent the standard deviation of the means. *p <.05, **p <.01, ***p <.001

Overall, each subject received 1,152 TMS pulses (288 pulses on each condition and each target site. TMS was applied to the target sites at the onset of the search display (i.e., stimuli‐onset TMS) in Experiment 1 (Figure 2a), while 500 ms prior to the search display onset (i.e., prestimuli TMS) in Experiment 2 (Figure 3a). Here, the order of target sites was counterbalanced across subjects, and operations were in accordance with published safety guidelines (Rossi et al., 2009; Wassermann, 1998).

### 2.4 Data analysis

Trials were excluded from analysis if the reaction times (RTs) fell outside the range of three standard deviations of mean RTs per condition (Experiment 1, 1.24%; Experiment 2, 1.03%), or the responses were incorrect (Experiment 1, 4.95%; Experiment 2, 2.52%). Using these criteria, there were 88.63 trials in Vertex‐uncued, 90.81 trials in Vertex‐cued, 89.75 trials in DLPFC‐uncued, and 91.06 trials in DLPFC‐cued condition for RTs analysis in Experiment 1; and there were 92.88, 92.75, 92.06, and 92.69 trials in Experiment. Additionally, we computed adjusted RTs by dividing the RTs by accuracy (adjRTs = RTs/accuracy) to evaluate overall search performance (Bardi et al., 2013; Bona et al., 2014; Wang et al., 2018). RTs, accuracy, and adjRTs were entered into a 2 (TMS Site: right DLPFC, Vertex) × 2 (Cueing: cued, uncued) × 2 (Distractor Color: white, colored) ANOVA with repeated measures. Bonferroni adjustment was used when conducting post‐hoc multiple comparisons.

## 3 RESULTS

### 3.1 Behavioral results of Experiment 1

As shown in Figure 2b, three‐way ANOVA of RTs revealed a significant main effect of Distractor Color [*F*_(1,15)_ = 11.340, *p* = .004, *η*_p_^2^ = 0.431; RTs_colored_ = 483 ms, RTs_white_ = 492 ms], indicating faster responses with salient distractors than with non‐salient distractors. Moreover, the ANOVA revealed an interaction effect between TMS Site and Cueing [*F*_(1,15)_ = 6.232, *p* = .025, *η*_p_^2^ = 0.294], indicating that the distractor location cueing effect differed when the rTMS was delivered to the right DLPFC and the Vertex. To check it, we averaged the RTs over the colored and white distractor condition and compared the cueing effect in the right DLPFC TMS and the Vertex TMS conditions. In consistent with the previous finding by Munneke et al. (2008), we found faster RTs in the cued condition than the uncued condition when rTMS was delivered to Vertex [*t*_(1,15)_ = −2.770, *p* = .014; RTs_cued_ = 483 ms, RTs_uncued_ = 501 ms; Figure 2c], confirming the role of the Vertex as a control site. When the rTMS was delivered to the right DLPFC, no cueing effect was observed [*t*_(1,15)_ = 0.300, *p* = .768; RTs_cued_ = 483 ms, RTs_uncued_ = 484 ms]. Moreover, we found RTs were fastened by the rTMS to the right DLPFC compared with the Vertex TMS in the uncued condition [*t*_(1,15)_ = −3.466, *p* = .003; RTs_DLPFC_ = 482 ms, RTs_Vertex_ = 501 ms], but not in the cued condition [*t*_(1,15)_ = 0.102, *p* = .920; RTs_DLPFC_ = 484 ms, RTs_Vertex_ = 483 ms]. No significant effects were found on accuracy (*p* > .05, Figure 2d).

To evaluate overall behavioral performance, we computed adjRTs (e.g., adjRTs = RTs/accuracy). Three‐way ANOVA of adjRTs revealed a main effect of Cueing [*F*_(1,15)_ = 7.489, *p* = .015, *η_p_* ^2^ = 0.333], and an interaction between TMS Site and Cueing [*F*_(1,15)_ = 6.202, *p* = .025, *η*_p_^2^ = 0.293]. Post hoc *t* tests on adjRTs revealed the same statistical results as in the RTs analysis. Specifically, adjRTs were faster in the cued condition than the uncued condition when rTMS was delivered to Vertex [*t*_(1,15)_ = −3.444, *p* = .004; adjRTs_cued_ = 512 ms, adjRTs_uncued_ = 544 ms], confirming the role of the Vertex as a control site. Compared with the Vertex stimulation, the right DLPFC stimulation fastened adjRTs in the uncued condition [*t*_(1,15)_ = −3.235, *p* = .006; adjRTs_DLPFC_ = 482 ms, adjRTs_Vertex_ = 501 ms]. In summary, these results showed that the rTMS delivered to the right DLPFC improved performance in the uncued trials, but not in the cued trials.

To examine whether the effect of right DLPFC TMS in the uncued condition was specific to trials where the distractor appeared in the contralateral or bilateral visual field to the rTMS site, we further analyzed the RTs for trials where the distractor was presented contralateral (i.e., left visual field, LVF) and ipsilateral to the right DLPFC (i.e., right visual field, RVF) separately. Here, two‐way ANOVA was performed with TMS Site (right DLPFC, Vertex) and Distractor Location (LVF, RVF) as factors. Analysis of RTs found a significant main effect of TMS Site [*F*_(1,15)_ = 11.653, *p = *.004, *η*_p_^2^ = 0.437]. Importantly, no interaction between TMS Site and Distractor Location was found [*F*_(1,15)_ = 1.308, *p* = .271, *η*_p_^2^ = 0.080], demonstrating that the DLPFC rTMS effect on the distractor was not specific to the left or right visual field.

### 3.2 Behavioral results of Experiment 2

In this experiment, online 10‐Hz rTMS was applied to the right DLPFC or Vertex 500 ms prior to the search display onset (Figure 3a). Procedures for data analysis were identical to Experiment 1. As shown in Figure 3b, three‐way ANOVA of RTs only revealed a main effect of Cueing [*F*_(1,15)_ = 34.060, *p* < .001, *η*_p_^2^ = 0.694], indicated as faster RTs when the distractor location was cued (480 ms) than when it was uncued (501 ms). No other effects were significant (*p* > .05). For accuracy (Figure 3c), three‐way ANOVA revealed an interaction effect between Cueing and Distractor Color [*F*_(1,15)_ = 8.741, *p* = .010, *η*_p_^2^ = 0.368]. Averaging the accuracies with the right DLPFC and Vertex stimulation, we found higher accuracy to a colored distractor than a white distractor in the uncued condition [*t*_(1,15)_ = 3.170, *p* = .006; accuracy_colored_ = 0.973, accuracy_white_ = 0.954; Figure 3d] but no significant difference in the cued condition [*t*_(1,15)_ = 0.875, *p* = .395; accuracy_white_ = 0.968, accuracy_colored_ = 0.964].

**FIGURE 4:**
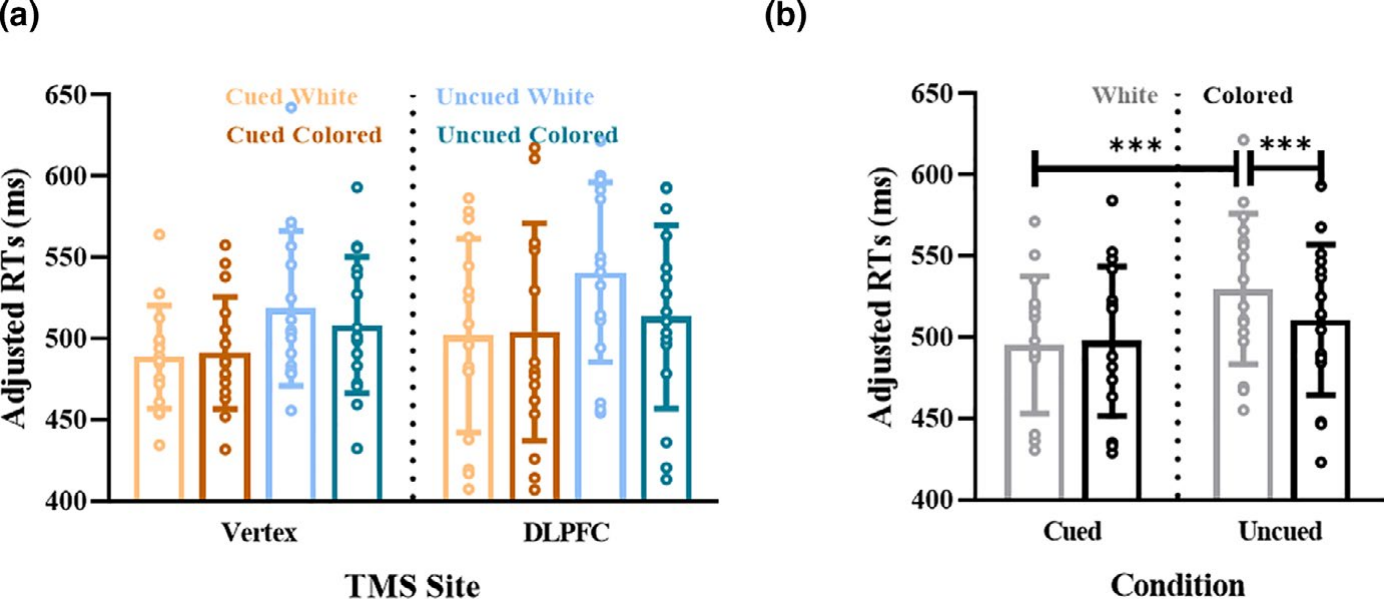
(a) Adjusted reaction times (adjRTs) per condition in Experiment 2. (b) adjRTs were plotted for trials in which the distractor was white or colored in the cued and uncued condition, respectively. adjRTs, RTs/accuracy. *p <.05, **p <.01, ***p <.001

Here, adjRTs showed the main effects of Cueing [*F*_(1,15_) = 21.233, *p* < .001, *η_p_* ^2^ = 0.586] and Distractor Color [*F*_(1,15_) = 8.755, *p* = .010, *η*_p_^2^ = 0.369], and an interaction effect between Cueing and Distractor Color [*F*_(1,15_) = 9.101, *p* = .009, *η*_p_^2^ = 0.378] (Figure 4a). No significant effect of the right DLPFC stimulation was found. Averaging across the right DLPFC and Vertex stimulation conditions, we found that adjRTs to colored distractor were faster than to the white distractor in the uncued condition [*t*_(1,15)_ = −3.896, *p* = .001; adjRTs_colored_ = 511 ms, adjRTs_white_ = 530 ms; Figure 4b], but no difference in the cued condition [*t*_(1,15)_ = 0.569, *p* = .578; adjRTs_colored_ = 498 ms, adjRTs_white_ = 495 ms]. In summary, the DLPFC stimulation delivered before the stimuli onset had no modulatory effect on the search task and the distractor location cue extinguished the effect of the salient distractor.

## 4 DISCUSSION

In the current study, we explored the role of the right DLPFC in distractor suppression using TMS. First, we found faster RTs in the cued trials compared to uncued trials, in line with earlier studies proposing that cueing the distractor location triggers an active inhibition at the cued location. Second, TMS over the right DLPFC eliminated such a cueing benefit reflected by faster RTs in the uncued trials and only at the search stimuli onset. Further analysis revealed that such TMS effect was derived from distractor inhibition at both visual fields. In addition, TMS over the right DLPFC showed no significant effect on distractor salience. Taken as a whole, we propose that the right DLPFC plays a key role in visuospatial distractor suppression and acts after the search stimuli are presented. Furthermore, it seems that the DLPFC contributes to distractor suppression triggered by the foreknowledge of distractor location instead of distractor salience.

A well‐known biased competition model assumes that overflowing stimuli compete for limited attentional resources (Bundesen, 1990; Desimone & Duncan, 1995). Both bottom‐up factors (e.g., stimulus salience) and top‐down factors (e.g., task relevance) bias sensory competition in favor of the most relevant input and further influence target‐processing (Awh et al., 2003; Itti & Koch, 2001; Noudoost et al., 2010; Posner, 1980; Theeuwes, 2010). On the one hand, salient‐but‐irrelevant information could automatically capture attention and drastically impair target‐processing (Broussard et al., 2006; Dorris et al., 2007). On the other hand, foreknowledge of the distractor location could reduce such impairment due to an active inhibition of the cued location (Chao, 2010; Munneke et al., 2008; Ruff & Driver, 2006). Our findings from the Vertex TMS were consistent with the basic tenet of the latter proposal.

Notably, the spatial cueing benefit was eliminated by stimuli‐onset TMS. Specifically, subjects' responses to the uncued trials were faster following the right DLPFC TMS than the Vertex TMS, indicating more efficient distraction filtering. With the Vertex TMS, responses were faster in the cued condition than in the uncued condition, confirming the role of the Vertex as a control site, which was used in various studies (Kalla et al., 2009; Kiyonaga et al., 2014; Wang et al., 2018). Theoretically, distraction filtering can be achieved by enhancing the representation of to‐be‐remembered items or by suppressing the representation of to‐be‐ignored items (Foxe & Snyder, 2011; LaBerge, 1995; Luck et al., 1997; Vanduffel et al., 2000). Based on this theory, a possible explanation is that TMS over the right DLPFC enhances the processing of the target. Accordingly, we would expect to observe a TMS effect whether the distractor location is cued or not cued. In fact, we did not reveal any TMS effect on the cued condition, which seems to be inconsistent with this account. Alternatively, TMS over the DLPFC may enhance the suppression of the distractor location. Indeed, the TMS effect exists only in the uncued condition makes a claim for the distractor suppression account. Additionally, we found significant reduction of RTs in both the distractor was presented in the left and right visual field, suggesting that the right DLPFC served to inhibit the distractor in both visual fields. Together, it seems the ability of suppressing distracting information is not visual field specific. Interestingly, no TMS effect was found if TMS was applied to the right DLPFC 500 ms prior to the search stimuli onset in both uncued and cued conditions. The finding is in line with a study by Tsotsos et al., (2008) which proposed that top‐down regulation appeared at least 100 ms after stimulus onset. In a later study, Kehrer et al., (2015) applied single‐pulse TMS over the right DLPFC or posterior parietal cortex at five time intervals (50, 100, 150, 200, 250 ms) after the probe display onset to examine the exact time course of spatial priming and they found significant TMS effects on priming effects only if the TMS was delivered 100 ms after the stimuli onset. Our finding of time‐dependent TMS effect surely demonstrated the advantage of online rTMS protocol which provides a potential advantage to test the exact timing of neural processing (Amassian et al., 1989; Beynel et al., 2019). Future research setting more time points and combining with high‐resolution techniques (e.g., electroencephalography) may be helpful to ascertain the exact time course. It has been proposed that top‐down inhibitory control over the distractor needs to selectively modulate the related neural circuit prior to the distractor onset (Noonan et al., 2018). In this study, the distractor location was validly cued in the cued condition, thus corresponding neural activities may differ from those in the uncued condition. Recently, an electrophysiological study by Heuer and Schubo (2019) found a smaller Pd component, a marker of active suppression, when the search display followed predictive than non‐predictive distractor cues. In other words, less suppressive processing of distractors was required if distractor location is known in advance. Therefore, we think that the foreknowledge of the distractor's location may reduce the need for distractor suppression, the DLPFC may contribute to this distractor suppression. Anyway, our results highlight a critical role of the right DLPFC in suppressing distracting information and contributing to it after stimuli presentation.

Apart from the location‐based cue, the distractor saliency randomly varied (white or colored) to trigger a feature‐based suppression, while the target remained white. Recently, several studies showed that foreknowledge of the saliency of salient‐but‐irrelevant distractors benefited behavior by actively suppressing this position (Gaspelin et al., 2015, 2017). In this study, colored distractors indeed led to better performance than the white ones in both experiments. Importantly, the distractor color and distractor cue interfered with each other when the TMS was delivered before the search stimuli onset. Specifically, colored distractor produced better behavioral performance (e.g., higher accuracy, faster adjusted reaction time) only when there was no prior knowledge of the distractor's location. These data showed priority of the location cue over the distractor color behaviorally. Importantly, TMS over the right DLPFC had no significant effect on the modulation of distractor saliency regardless of the timing of the TMS application. We think that this might be related to attention networks for spatial and non‐spatial features. Giesbrecht et al., (2003) found that portions of the frontoparietal network were commonly activated to the location and color cues of a target, but dorsal areas within the network were more active when orienting attention to locations than colors, indicating that these regions were specific for controlling visuospatial attention. Here, the TMS was delivered to the right DLPFC, as a dorsal area, may be more important for location‐based distractor suppression.

Our study applied online rTMS (3 pulses at 10 Hz) which has been proved to be an effective protocol to modulate cortical activity (Lega et al., 2019; Saad & Silvanto, 2013). We used a fixed intensity of 30% of the maximum stimulator output, which was much lower than previous protocols‐60% or 65% of the maximum stimulator output (Kiyonaga et al., 2014; O'Shea et al., 2004; Pitcher et al., 2007, 2008). Recently, Zmeykina et al. (2020) measured weak rTMS‐induced EEG signals and found that weak rTMS with the intensity of about only 16.8 ± 2% and 23.9 ± 2.5% of the maximum stimulator output could reliably induce electrophysiological effects. Combined with less discomfort and rTMS effectiveness with lower intensity, we believe the rTMS with low intensity may serve as a good choice for future TMS studies. Still, the present study has some limitations. First, the Vertex, which served as the control site in the present study, corresponds to the supplementary motor area (SMA) in at least 50% of participants (Okamoto et al., 2004). Considering that SMA, conjunction with the inferior frontal cortex, is a crucial hub of the proactive and reactive inhibition network (Aron et al., 2004, 2014), sham TMS may serve as an alternative control condition. The sham TMS aims to mimic the auditory and/or somato‐sensory effects of active TMS without actual stimulation of the brain. However, it still differs from the active TMS in some aspects, for example, participants' nervousness. Second, this study did not find TMS effects of the DLPFC on feature‐based suppression, future studies may ascertain potential contribution of other brain areas, such as the posterior parietal cortex, to feature‐based suppression (Schenkluhn et al., 2008; Zanto & Rissman, 2015).

In summary, we used the online rTMS protocol to explore the role of the right DLPFC in distractor suppression. Here, we replicated existing findings that the competing distractor was suppressed when its location or salient feature was known in advance. Importantly, the right DLPFC seems to be selectively recruited for visuospatial distractor suppression and act after the search stimuli are presented. In addition, it seems that the right DLPFC is more involved in distractor suppression triggered by foreknowledge of distractor location rather than by distractor salience, consistent with spatial attention network theme.

## CONFLICT OF INTEREST

The authors declare no conflict of interest.

## AUTHOR CONTRIBUTIONS

Z.J.: designed the study; K.X., X.N. and D.J.: collected the data; K.X., X.N. D.J., and J.Z.: analyzed the data; Z.J., K.X., L.L.: wrote the manuscript.

### Peer Review

The peer review history for this article is available at https://publons.com/publon/10.1111/ejn.15164.

## Data Availability

All data are available upon reasonable request.
